# Optimal slice thickness for object detection with longitudinal partial volume effects in computed tomography

**DOI:** 10.1002/acm2.12005

**Published:** 2016-11-23

**Authors:** Pascal Monnin, Nicolas Sfameni, Achille Gianoli, Sandrine Ding

**Affiliations:** ^1^ Haute Ecole de Santé Vaud (HESAV) Filière TRM Lausanne Switzerland; ^2^ Institute of radiation physics (IRA) University Hospital of Lausanne (CHUV) Lausanne Switzerland

**Keywords:** artifacts and distortion, computed tomography, contrast, noise, spatial resolution

## Abstract

Longitudinal partial volume effects (z‐axial PVE), which occur when an object partly occupies a slice, degrade image resolution and contrast in computed tomography (CT). *Z*‐axial PVE is unavoidable for subslice objects and reduces their contrast according to their fraction contained within the slice. This effect can be countered using a smaller slice thickness, but at the cost of an increased image noise or radiation dose. The aim of this study is to offer a tool for optimizing the reconstruction parameters (slice thickness and slice spacing) in CT protocols in the case of partial volume effects. This optimization is based on the tradeoff between axial resolution and noise. For that purpose, we developed a simplified analytical model investigating the average statistical effect of *z*‐axial PVE on contrast and contrast‐to‐noise ratio (CNR). A Catphan 500 phantom was scanned with various pitches and CTDI and reconstructed with different slice thicknesses to assess the visibility of subslice targets that simulate low contrast anatomical features present in CT exams. The detectability score of human observers was used to rank the perceptual image quality against the CNR. Contrast and CNR reduction due to *z*‐axial PVE measured on experimental data were first compared to numerical calculations and then to the analytical model. Compared to numerical calculations, the simplified algebraic model slightly overestimated the contrast but the differences remained below 5%. It could determine the optimal reconstruction parameters that maximize the objects visibility for a given dose in the case of *z*‐axial PVE. An optimal slice thickness equal to three‐fourth of the object width was correctly proposed by the model for nonoverlapping slices. The tradeoff between detectability and dose is maximized for a slice spacing of half the slice thickness associated with a slice width equal to the characteristic object width.

## 1 Introduction

The detection of thin objects with low contrast in computed tomography (CT) is crucial to differentiate anatomical features with close densities and distinguish tumors. Consequently, studies regularly investigate the best settings to improve object detectability for different tasks.^1^, ^2^, ^3^ Contrast and detection of small objects can be dramatically lowered when spatial resolution along the longitudinal *z*‐axis is not adapted to object thickness. *Z*‐axial (longitudinal) partial volume effect (PVE) is unavoidable for subslice objects (objects thinner than the slice width). It can also randomly affect those smaller than twice the slice thickness according to their fraction contained within the slice. Object undersampling in the longitudinal direction introduces quantitative biases in Hounsfield units (HU), and adversely lowers the contrast.^4^, ^5^, ^6^ Contrast loss introduced by *z*‐axial PVE is not routinely considered and is often overlooked in clinical practice since it can be reduced using thin‐slice scanning. Reconstructing thin slices is, however, associated with small pitches, high noise, or increased dose. Nevertheless, tradeoffs between axial resolution and noise can be envisaged to optimize CT protocols for an envisaged clinical application.^7^ Previous studies have tested various protocols to find the slice thickness,^8^, ^9^, ^10^ slice spacing,^11^, ^12^ or interpolation algorithm^13^ that would give the best compromise between *z*‐axial resolution and noise. Although the physical relationships between axial resolution and noise in CT are long known, the stochastic influence of *z*‐axial PVE on the contrast of small objects has never been taken into account. Clinical medical physicists have, however, to deal with partial volume effects for (1) optimizing the objects detectability, and (2) minimizing the dose, which is especially important in CT.^1^, ^14^

The goal of this study is to offer a tool for optimizing the reconstruction parameters (slice thickness and slice spacing) in CT protocols for a given detection task involving *z*‐axial PVE. The *z*‐axial PVE is a complex phenomenon that implies statistical variations due to the object positioning within the slice, and a simple signal averaging in the slice volume is not adequate for predicting contrast attenuation and detection jeopardizing. The exact determination of an expected contrast‐to‐noise ratio (CNR) with *z*‐axial PVE requires numerical computation based on the object and slice *z*‐profiles, and the optimization problem is therefore difficult to resolve in clinical practice. This study develops an approximate analytical model that allows an easy determination of the reconstruction parameters corresponding to the highest CNR or lowest dose for cross‐sectional imaging of thin objects. It uses a quantitative relationship between noise, axial resolution, and dose to establish the optimal reconstruction parameters that maximize detection tasks with *z*‐axial PVE. It averages out the stochastic object positioning within the slices, as a function of object size and slice thickness. First, it is tested using a standard image quality phantom made of well‐calibrated objects, shapes, and length. Then, it is compared to the exact solution of the problem determined by numerical computation. The contrast, CNR, and detectability of thin objects of different lengths are assessed with *z*‐axial PVE for different imaging conditions (pitch, noise, and slice thickness). A reader study confirms that the detectability of subslice targets is CNR dependent, as expected from previous results obtained for larger objects,^2^, ^15^ and validates the relevance of the algebraic model for optimal scanning parameters choice and dose minimization.

## 2 Materials and methods

### 2.A Theory

#### 2.A.1 Exact calculation

When a voxel contains different densities, the resulting signal within the voxel is the average of the signals from the different materials. The signal (HU) for an object located partially in a slice is weighted according to its fraction within the slice. Accordingly, the contrast *C(z)* of such objects may vary depending on their *z*‐position in a slice. It is given by the convolution product between the longitudinal contrast profile *C*_0_ *(z)* of the object and the slice sensitivity profile (*SSP(z*)), the point spread function (PSF) of the imaging system along the *z*‐axis

(1) $$C\left(z\right)=C_{0}\left(z\right)*SSP\left(z\right)=\int_{-\infty }^{\infty }C_{0}\left(\zeta \right)SSP\left(z-\zeta \right)d\zeta .$$

For the particular case of a cylindrical object of length *L* and CT number *μ* located in a background of CT number *μ*_0_,

(2) $$\begin{array}{cc} C_{0}\left(z\right) & =\left(\mu -\mu_{0}\right)\cdot rect\left(\frac{z}{L}\right),\;\text{with the rectangular function}\;\\ & rect\left(x\right)=\left\{\begin{array}{cc} 1, & x\in [-0.5;0.5]\\ 0, & otherwise\; \end{array}\right.. \end{array}$$

The statistical expected object contrast $\bar{C}$ is given by the value of *C(z)* averaged over all the possible *z*‐positions within the effective slice thickness *T*_*e*_

(3) $$\bar{C}=\frac{1}{T_{e}}\int_{-T_{e}/2}^{T_{e}/2}C\left(z\right)dz.$$

The effective slice thickness (*T*_*e*_) depends on the slice sensitivity profiles (SSP) extent. The full width at half maximum (FWHM) is used for *T*_*e*_ in usual regulations.^16^, ^17^ The SSP is, however, not fully characterized by its FWHM. The longitudinal signal spread will depend on its entire shape: long tails will degrade the *z*‐resolution more than a close to rectangular SSP, even if both have the same FWHM.^18^ For a more precise characterization of the longitudinal signal spread, the mean width of the SSP was used as a measure of the effective slice thickness in this study.

#### 2.A.2 Simplified algebraic model

A simplified algebraic expression for the expected contrast can be used to provide an understanding relationship between the quantitative parameters involved in the contrast loss. This is done by approximating the SSP with a rectangular function of height equal to one, and width equal to the effective slice thickness (*T*_*e*_). The object contrast profile *C*_0_(*z*) can also be approximated by a rectangular function of height *C*_0_ and width *L*. In this study, *L* represents the characteristic object length—for example, $\frac{\pi }{4}d$ for a sphere of diameter *d*. Considering the slice which contains the largest proportion of the object (the slice with the highest object contrast), the object length in this slice can be between *L/2* and min(*T*_*e*_, *L*). Considering *λ* = *L/T*_*e*_ the ratio between the characteristic object length and the effective slice thickness, *z*‐axial PVE may occur only if *λ* < 2. Two cases have to be further distinguished: 1) the object is thinner than *T*_*e*_ (0 < *λ* ≤ 1), and *z*‐axial PVE is unavoidable; 2) the object width is between one and two‐times *T*_*e*_ (1 ≤ *λ* < 2), and *z*‐axial PVE will occur stochastically, depending on the object fraction within the slice.

**Figure 1:**
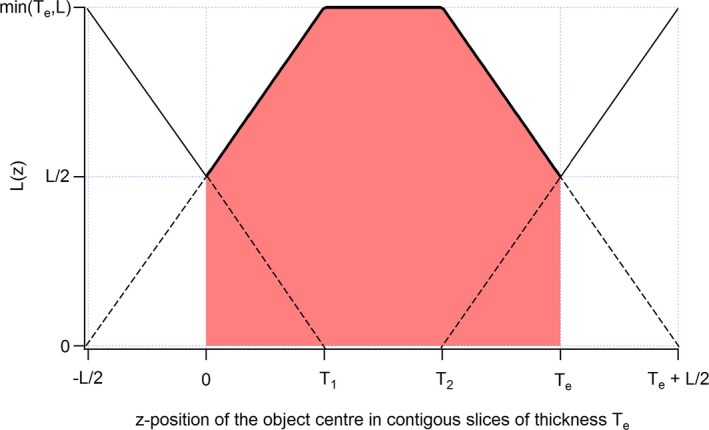
Maximal length *L(z)* of a rod contained in nonoverlapping slices of effective thickness *Te* as a function of its *z*‐position. The *z*‐axis position of the object is here defined from the central z‐point of the object. *T*_*1*_ = min(*T*_*e*_, *L*)−*L*/2 and *T*_*2*_ = max(*T*_*e*_, *L*) −*L*/2.

Figure 1 shows the convolution product between the two rectangular functions of lengths *L* and *T*_*e*_. The *z*‐position of the object is here defined from the central *z*‐point of the object (since objects may have different lengths) relative to the edge of the slice (positioned at z = 0). The plain line in Fig. 1 shows the object length *L*(*z*) in the slice containing the largest object length, as a function of the object *z*‐position, for nonoverlapping slices. The position of this slice covers the red area. The statistical expected object length $\bar{L}$ contained in this slice is an average over all possible *z*‐positions of the object and is given by the average height of the trapezoidal red area in Fig. 1,

(4) $$\bar{L}=\frac{1}{T_{e}}\int_{-T_{e}/2}^{T_{e}/2}L\left(z\right)dz=L\left(1-\lambda /4\right).$$

Still considering the slice with the largest object length (slice with the highest contrast), the expected object contrast $\bar{C}$ will be weighted by the expected object fraction within the effective slice width ($\bar{L}/T_{e}$). For a homogeneous object of CT number *μ* in a background of CT number *μ*_0_, this gives

(5) $$\bar{C}=\left(\mu -\mu_{0}\right)\frac{\bar{L}}{T_{e}}=\lambda \left(1-\lambda /4\right)\left(\mu -\mu_{0}\right).$$

Equation (5) shows that *z*‐axial PVE reduces the expected object contrast by the factor *λ*(1 − *λ*/4) ≤ 1 (for *λ* ≤ 2). The highest and lowest contrasts possible, always considering the slice with the highest contrast, occur when the entire object and half object are contained in the slice. They are given in eqs. (6a) and (6b), respectively.

(6a) $$C_{\max}=\lambda \left(\mu -\mu_{0}\right)$$

(6b) $$C_{\min}=\frac{\lambda }{2}\left(\mu -\mu_{0}\right)$$

Equations (4) and (5) are valuable for nonoverlapping slices only. For a slice spacing *αT*_*e*_ (*α* ≤ 1), the partial overlap between adjacent slices leads to a decrease in the length over which the expected object length and contrast must be integrated (red area in Fig. 1). The integration length is then reduced to (2*α* − 1)*T*_*e*_ instead of *T*_*e*_. The expected object length and contrast for 0.5 < *α* ≤ 1 are given in eqs. (7) and (8), respectively.

(7) $$\bar{L}=\frac{L\left(\alpha -\lambda /4\right)-T_{e}{\left(1-\alpha \right)}^{2}}{2\alpha -1}$$

(8) $$\bar{C}=\frac{\lambda \left(\alpha -\lambda /4\right)-{\left(1-\alpha \right)}^{2}}{2\alpha -1}\left(\mu -\mu_{0}\right)$$

**Figure 2:**
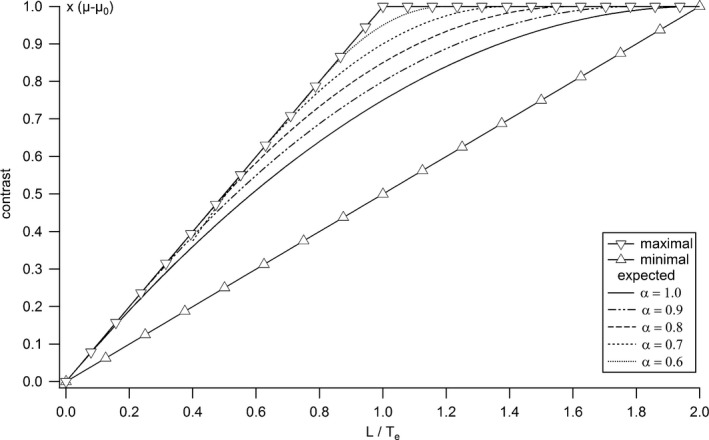
Maximal, minimal, and expected contrasts as functions of the ratio between object and slice thicknesses, calculated from the analytical model for different slice spacing.

As expected, the particular case *α* = 1 (non‐overlapping slices) in eqs. (7) and (8) leads to eqs. (4) and (5), respectively. For *α* between 0.5 and 1, the smaller the slice spacing the closer the expected contrast curve approaches the highest contrast possible (Fig. 2). Additionally, the range in which *z*‐axial PVE can occur is determined by *λ* < 2*α*, and decreases with *α*. A value of *α* equal to 0.5 will give the highest expected contrast for a given slice thickness. Reducing *α* to less than half is useless.

### 2.B Image acquisition

**Figure 3:**
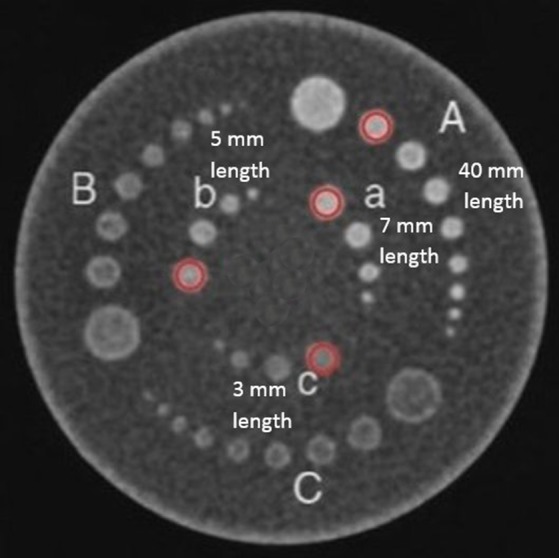
CT slice of the CTP515 module of the Catphan 500 phantom (circled: rods considered in the study).

A cylindrical Catphan 500 phantom (The Phantom Laboratory, Salem, NY) of 20 cm diameter was used to assess the visibility of thin targets that simulate low‐contrast anatomical features present in CT exams. A GE LightSpeed VCT scanner (GE Healthcare, Waukesha, WI) was used to acquire the CTP515 module of the phantom that contains six groups of rods of low contrast (A, B, C, a, b, c in Fig. 3) arranged in two concentric circles with a homogeneous background. Only rods A, a, b, and c of 40, 7, 5, and 3 mm length, respectively, and of 9 mm diameter were considered in our study (circled rods in Fig. 3). These four rods have the same density. Rod A of length 40 mm is not subject to *z*‐axial PVE and was therefore used for reference measurements of object signal (*μ*) or contrast (*μ*−*μ*_0_).

The phantom was placed in the phantom holder supplied by the manufacturer on the CT table, and aligned to the isocenter using the alignment lasers. Orthogonal scout views were used to control the correct positioning of the phantom. All acquisitions were performed at 120 kV, with a rotation time of 1 s, a 8 × 1.25‐mm collimation, and a scan field of view (SFOV) of 25 cm in diameter, using the helical mode with two pitch values, 0.875 and 1.675, and the automatic current modulation with three noise indexes: 5, 10, and 15. For each protocol (Table 1), the data were reconstructed by filtered backprojection (FBP) with a standard abdominal reconstruction kernel into three nonoverlapping slices of 1.25, 2.5, and 5 mm. The CTDI_vol_ displayed by the scanner for the 18 protocols are reported in Table 1. Ten acquisitions of each protocol were performed, slightly moving the phantom along the longitudinal *z*‐axis between each acquisition, while the slices were always reconstructed from the same *z*‐position. This created variability in the *z*‐position of the rods and induced stochastic realizations of *z*‐axial PVE.

**Table 1:** Acquisition and reconstruction parameters and CTDI_vol_ of CT protocols

Protocol	Noise index	Pitch	CTDI_vol_ (mGy)	Slice thickness (mm)
1/2/3	5	0.875	106.5	1.25/2.5/5.0
4/5/6		1.675	55.6	1.25/2.5/5.0
7/8/9	10	0.875	55.2	1.25/2.5/5.0
10/11/12		1.675	26.5	1.25/2.5/5.0
13/14/15	15	0.875	24.5	1.25/2.5/5.0
16/17/18		1.675	11.8	1.25/2.5/5.0

### 2.C Effective slice thicknesses (T_e_)

The 0.28‐mm tungsten carbide bead of the CTP528 module of the Catphan phantom was used as a subpixel point source to determine the slice sensitivity profiles (SSP) of the CT slices along the *z*‐direction.^19^ The bead was scanned using the lowest noise index (NI = 5) and two pitches of 0.875 and 1.675. Slices of the bead were reconstructed with the same parameters as the CTP515 module, by FBP into the three slice thicknesses of 1.25, 2.5, and 5 mm, using the standard abdominal reconstruction kernel. Determining the SSP requires a thin *z*‐oversampling with strong overlap between slices. The images of the bead were therefore reconstructed with a slice spacing equal to one‐tenth of the slice thickness (90% overlap). A circular region of interest (ROI) was placed within the slice of the bead with the highest signal, and propagated through all adjacent slices. The oversampled signal value of the bead in the ROI was plotted against the slice location producing the SSP. Because the bead is subslice sized, no correction for the bead size was applied on the measured SSP.

### 2.D Contrast, noise, and contrast‐to‐noise ratio (CNR)

For each acquisition, the slice with the highest object contrast was chosen and a circular ROI placed within each of the four cylinders gave the mean pixel value (HU) for each of them (Fig. 3). Another circular ROI of 50 pixels in diameter positioned in the background at the center of the phantom was used for mean background signal (*μ*_*0*_) and noise measurements. The contrast for each cylinder corresponds to the absolute difference between the cylinder and the background HU values, averaged over the 10 acquisitions. Rod A of length 40 mm was not subjected to *z*‐axial PVE and its contrast, equal to (*μ*−*μ*_0_) for all protocols, was taken as the reference contrast used to normalize all rod contrasts. The measured mean contrasts were then compared to the expected contrasts obtained with numerical calculations of eq. (3), and with the approximate analytical eq. (5). The standard deviation of the pixel values in the background ROI (*σ*_0_) provided the noise level. The expected CNR ($\bar{CNR}$ ) for each cylinder was then calculated using eq. (9).

(9) $$\bar{CNR}=\frac{\bar{C}}{\sigma_{0}}$$

### 2.E Subjective image quality evaluation

Phantom scoring was used to rank the perceptual image quality against the CNR, whose value is reduced by *z*‐axial PVE. The detection test was performed by four human observers on an Eizo RadiForce R22 diagnostic screen whose greyscale display function was calibrated to the Dicom Part 14 standard. The standard display window W/L = 400/40 for routine abdominal examinations was used. For each CT series, only the image with the highest contrast was shown to the observers. The 180 images (10 acquisitions × 18 protocols) were randomly displayed one at a time to each observer without any parameter displayed. Display time per image was free but cumulated observation time was limited to 45 min per observer per day. Visualization of the four cylinders A, a, b, and c (Fig. 3) was noted on a three level scale, 0, 0.5, and 1, corresponding to a nonvisible, partially visible, and totally visible object. The average image quality score for the 10 acquisitions of a given protocol was established for each cylinder and observer. The agreement between the four observers was assessed with the Fleiss’ kappa test using the R statistical software (R Development Core Team).

## 3 Results

### 3.A Subjective image quality evaluation

**Figure 4:**
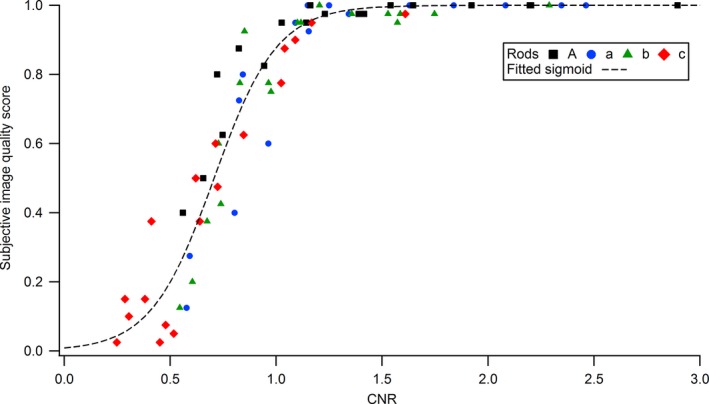
Relationship between the subjective image quality scores averaged over the four observers and the measured CNRs for the four rods.

The concordance level of subjective image scoring for the four human observers (*κ*) was 0.504, and indicated an adequate inter‐reader agreement according to the scale of Landis and Koch.^20^ The average image quality scores of the four observers were therefore considered for the 18 protocols and the four cylinders. The average scores were linked to their corresponding object CNRs with a fitted sigmoid curve (Fig. 4).

### 3.B Noise level

For a given CT geometry, the number of photons detected per voxel is proportional to the CTDI_vol_ and voxel volume (and thus slice thickness). For a quantum limited system, noise statistics in the images is Poissonian and noise (standard deviation of pixel values) is inversely proportional to the square root of the product CTDI_vol_ by slice thickness (*T*) for a fixed spatial resolution in the slice plane.^21^ Standard deviation of HU (*σ*_0_) measured in the homogeneous circular ROI at the center (background) of the phantom were thus fitted using a log–log linear function of the product CTDI_vol_ by slice thickness (*T*),

(10) $$\log\left(\sigma_{0}\right)=a-b\cdot \log\left(CTDI_{vol}\cdot T\right).$$

**Figure 5:**
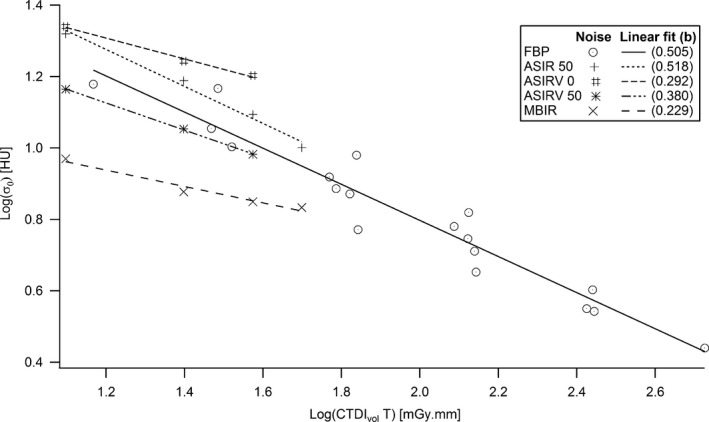
Noise at the center of the phantom as a function of the product between CTDI_vol_ and slice thickness.

Fit coefficients *a* = 1.808 HU and *b* = 0.505 HU/(mGy mm) were obtained with a Pearson's correlation coefficient of −0.965 (Fig. 5). Equation (10) was also tested for other noise data obtained on scanners equipped with different iterative reconstruction (IR) algorithms. Fit coefficients *b* equal to 0.229, 0.292, 0.380, and 0.518 were obtained with a high degree of linear correlation for the IR algorithms model‐based iterative reconstruction (MBIR), ASIRV (adaptive statistical iterative reconstruction‐V) 0, ASIRV 50, and ASIR (adaptive statistical iterative reconstruction) 50, respectively (Fig. 5). The square root dependence of noise with the dose and the assumption that quantum noise is dominant in the slices is confirmed for FBP and ASIR 50 reconstruction algorithms (fitted coefficient *b* very close to 0.5 in eq. (10)).

### 3.C Contrast reduction factor

**Figure 6:**
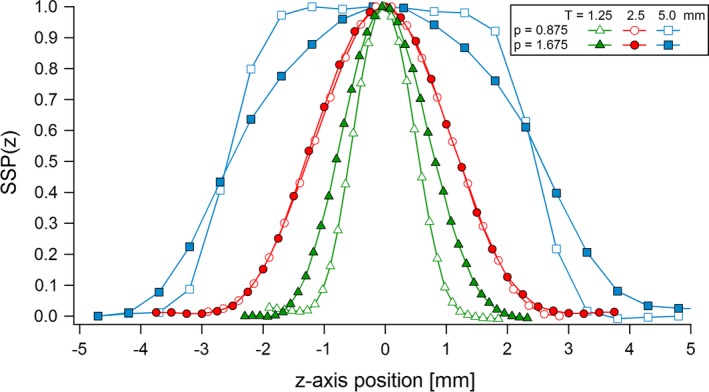
SSP measured for the three slice thicknesses and two pitches used in the study.

**Figure 7:**
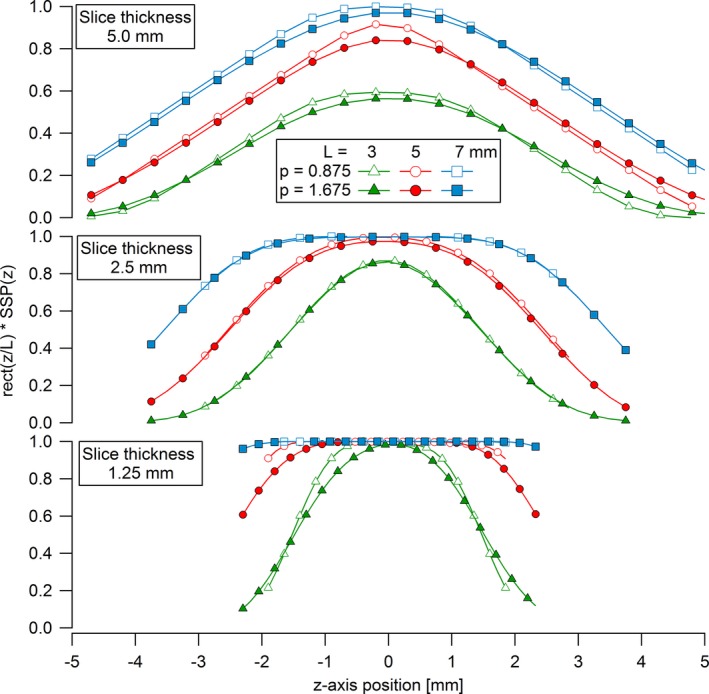
Numerical calculations of normalized contrasts for rods of lengths *L* = 3, 5, and 7 mm as functions of their *z*‐position for the three slice thicknesses and two pitches used in the study.

All the FWHMs and mean widths of the measured SSP (Fig. 6) given in Table 2 were close to the corresponding slice thicknesses, with differences smaller than ± 0.11 mm. The only exception is for the 1.25 mm slice with a pitch of 1.675, whereas effective widths of 1.63 and 1.69 mm were measured. Figure 7 shows the numerical calculations of the normalized contrast profiles *C*(*z*)/(*μ* − *μ*_0_) obtained with eq. (1) for the different combinations of cylinders lengths and slice thicknesses. The expected normalized contrasts $\bar{C}/\left(\mu -\mu_{0}\right)$ calculated with eq. (3) from the curves in Fig. 7 were taken as the (exact) reference values for the expected contrast reduction factors (Table 2).

**Table 2:** FWHM and mean width of the SSP, and expected contrast reduction factor obtained from eq. (2) for the three rods

Slice thickness (mm)	Pitch	FWHM of the SSP (mm)	Mean width of the SSP (mm)	Expected contrast reduction factor		
				Rod a (*L *= 3 mm)	Rod b (*L *= 5 mm)	Rod c (*L *= 7 mm)
1.25	0.875	1.15	1.20	0.988	0.999	0.999
	1.675	1.63	1.69	0.959	1.000	1.000
2.5	0.875	2.49	2.56	0.769	0.962	0.998
	1.675	2.52	2.61	0.763	0.942	0.995
5.0	0.875	5.04	5.00	0.493	0.733	0.893
	1.675	5.09	4.96	0.470	0.709	0.869

**Figure 8:**
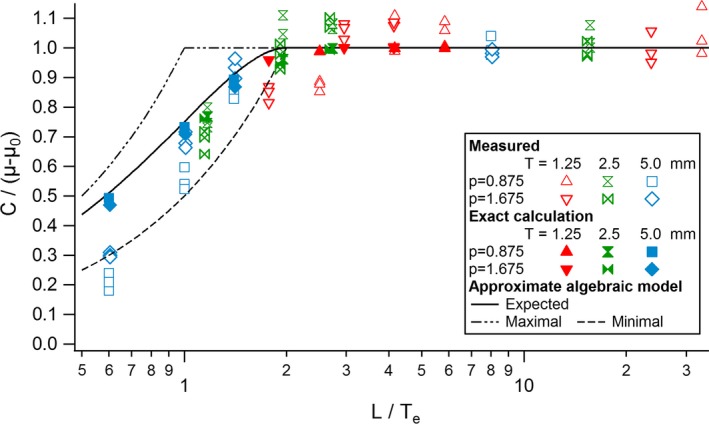
Expected normalized contrasts of the rods measured on the slice with the highest object contrast compared to the (exact) numerical calculations and the algebraic model.

The contrast of rod A (not subject to *z*‐axial PVE) was constant for all the protocols and equal to the nominal contrast value of 8 HU. The contrasts of rods *a*,* b*, and *c* measured on the slices and averaged over the 10 different acquisitions were thus normalized by 8 HU. This gave the corresponding contrast reduction factors for the different ratios *λ* = *L/T*_*e*_. The measured contrast reduction factors were then compared to their corresponding theoretical values obtained (1) with numerical calculations of eq. (3), and (2) with the simplified algebraic model (eq. (5); Fig. 8). All the measured contrasts are at their nominal value for *λ* ≥ 2, where no *z*‐axial PVE occurs. But as expected, they fall off as a function of *λ* below 2, due to *z*‐axial PVE. The variability in the averaged contrasts measured for a given *L*/*T*_*e*_ ratio shows statistical variability in *z*‐axial PVE between the data acquired with the three different noise indexes. Averaged normalized contrasts measured on the slices are consistent with the calculated expected contrast reduction factors given in Table 2. An exception happened for the smallest *L*/*T*_*e*_ ratio (*λ* = 0.6) for which measured contrasts were up to 50% lower than the theoretical values obtained from numerical and analytical calculations. Compared to the numerical calculation, the simplified algebraic model slightly overestimates the contrast but the differences always remain below 5%.

### 3.D Contrast‐to‐noise ratio (CNR)

The expected contrast from the simplified model (eq. (8)) and the noise level from eq. (10) were used to determine the theoretical expected CNR ($\bar{CNR}$ ) of the rods as a function of the ratio *λ* = *L*/*T*_*e*,_

(11) $$\bar{CNR}\left(\lambda \right)=10^{-a}{\left(CTDI_{vol}\cdot L\right)}^{b}\frac{\bar{C}\left(\lambda \right)}{\lambda^{b}}.$$

**Figure 9:**
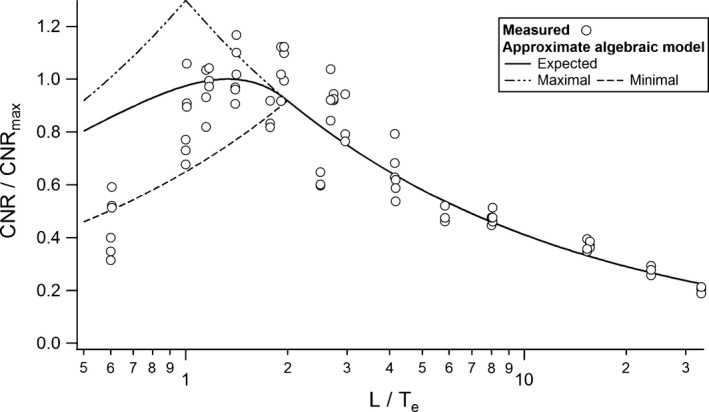
Expected CNR normalized to its maximal possible value as a function of the ratio *L*/*Te*.

The measured CNR agree with the values predicted by eq. (11), except for the smallest *L*/*T*_*e*_ ratio, as measured contrasts were up to 50% lower than the theoretical values (Fig. 9). Equation (11) was used to analyze the slice thickness required to restore the CNR to an optimal value for a given CTDI. The expected CNR is the highest for *λ* equal to

(12a) $$\lambda {|}_{{\bar{CNR}}_{\max}}=\frac{2}{2-b}\left(\alpha \left(1-b\right)+\sqrt{\alpha^{2}-b\left(2-b\right)\left(2\alpha -1\right)}\right),$$

that reduces to eq. 12b for slices reconstructed by FBP (*b* = 0.5)

(12b) $$\lambda {|}_{{\bar{CNR}}_{\max}}=\frac{2}{3}\left(\alpha +\sqrt{4\alpha^{2}-6\alpha +3}\right).$$

**Figure 10:**
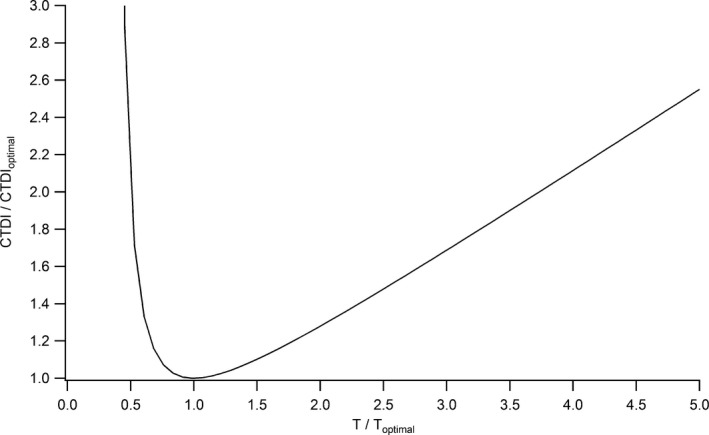
CTDI variation as a function of the ratio between the chosen and optimal slice thicknesses for maintaining a constant CNR (nonoverlapping slices, *α* = 1).

**Figure 11:**
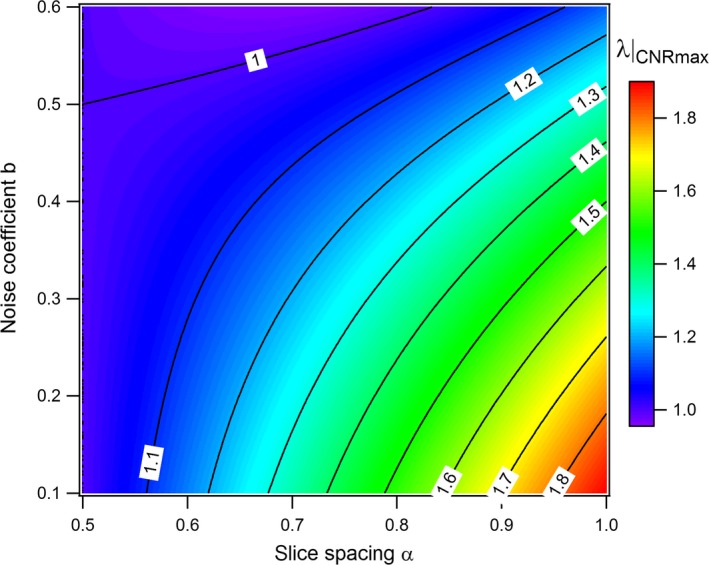
*L*/*T*_*e*_ ratio (*λ*) for maximizing the CNR as a function of slice spacing *α* and noise coefficient *b*.

The measured CNR shown in Fig. 9 reach a peak for *λ* = 4/3, as predicted by eq. (12b) for nonoverlapping slices (*α* = 1). The choice of a slice thickness *T* = 0.75*L* maximizes the expected visibility of objects of length *L* in this case. For partially overlapping slices (*α* < 1), the optimal *λ* decreases with the slice spacing and reaches 1 for *α* = 0.5. This implies that the optimal slice thickness increases with the slices overlap from 0.75*L* up to *L* for 50% overlap. Figure 10 shows the iso‐CNR curve based on eq. (11) as a function of the ratio between the chosen and optimal slice thickness. It indicates how a suboptimal choice in slice thickness should be compensated by an increase in CTDI for maintaining a given CNR in the case of *z*‐axial PVE. A choice of a slice two times thicker than the optimal thickness would, for example, necessitate a dose increase of 28% to hold a constant CNR for nonoverlapping slices. Figure 11 gives the optimal parameter *λ* as a function of slice spacing and noise behavior (parameter *b*). For nonoverlapping slices reconstructed with iterative algorithms, the optimal slice thickness is thinner than 0.75*L* with *T*_*e*_/*L* = 0.57, 0.60, 0.65, and 0.75 for MBIR, ASIRV 0, ASIRV 50, and ASIR 50, respectively.

## 4 Discussion

Decreasing the slice thickness restores axial resolution and contrast in the case of *z*‐axial PVE, but adversely increases image noise. An optimal slice thickness dependent on the object size is therefore expected to maximize the detection of a thin object jeopardized by *z*‐axial PVE. But, the knowledge of the optimal reconstruction parameters requires numerical computation based on the complex object *z*‐shape and SSP. Optimization of slice thickness is thus not routinely performed in clinical practice. A simplified analytical model that determines easily the optimal slice thickness and spacing that maximizes the CNR and detection for a given CTDI was proposed (eqs. (5), (8)). It approximates the SSP and the object *z*‐profile by two rectangular functions of widths equal to the effective slice thickness (*T*_*e*_) and the characteristic object length (*L*). The characteristic length of the object *L* and noise coefficient *b* are the two parameters determining the optimal slice thickness and spacing. For nonoverlapping slices with a perfect quantum noise behavior (FBP and ASIR 50), the model proposes an optimal slice thickness *T* = 0.75*L*. The tradeoff between detectability and dose is maximized by a slice spacing of half a slice thickness equal to the characteristic object length. The model determined the expected object contrast loss due to *z*‐axial PVE with less than 5% error compared to numerical calculations. An exception concerned the smallest *L*/*T*_*e*_ ratio (*λ* = *L*/*T*_*e*_ = 0.6), for which differences in expected contrasts of up to 50% were observed. The origin of this bias remained unknown, but it affected only the precision of the model for very strong *z*‐axial PVE.

Phantom scoring was used to study the detectability of subslice targets and rank the perceptual image quality against the CNR, which is commonly used as a figure of merit for image quality assessment. This assessed the negative impact of *z*‐axial PVE on the detection of low‐contrasted objects, and is consistent with results obtained in a previous study.^22^ A straightforward relationship between CNR and detection was confirmed, as expected by the Rose model for the considered conditions (objects of low contrast in homogeneous noisy background).^23^ Detectability and CNR of subslice targets followed a sigmoidal function, in agreement with Weber's law. This law predicts the detection probability of a given object as a function of the stimulus magnitude on the image follows a psychometric curve described by the integral of the normal probability curve.^24^

In our article we used cylindrical rods to represent for instance tumors of different sizes. If the rods were placed at different orientations it would have change two aspects. Indeed, a change in object orientation relative to the longitudinal CT *z*‐axis reverses to a change in the characteristic longitudinal object length *L*. It will also affect the sharpness of the object boundaries. These two effects are considered in our model through the calculation of the characteristic object length (*L*), defined as the average width of the object *z*‐profile. A modification in the orientation of the objects would shorten *L*, and thereby the value of the variable *λ* = *L*/*T*_*e*_ considered in our study. The optimal slice thickness given by the model would thus change with the object orientation in the same way it changes with the object length or size.

Given the simplicity of the objects’ shape and phantom's geometry used in our study, the algebraic model was tested under experimental conditions that are as close as possible to the assumptions of the model (objects with rectangular *z*‐profiles in homogeneous noisy background). To be validated, the model needed standardized and reproducible object shapes. A lower degree of accuracy is thus expected in a clinical environment, where tumors have complex shapes and the patient images contain anatomical noise. Nevertheless, results from this study suggest that the model provides a reasonable guidance to optimize reconstruction parameters in clinical protocols. For example, the detection of rod *c* in protocol 3 was compromised by *z*‐axial PVE (detection score = 0.375, CNR = 0.41). This result was expected since the parameter *λ* was at a suboptimal value of 0.6 due to a slice too thick (5 mm) compared to the object length (3 mm). Switching to protocol 5, the detection score of rod *c* could be dramatically improved to 0.95 (CNR = 1.18) while reducing the CTDI_vol_ by half (from 106.5 to 55.6 mGy). Protocol 5 used a slice thickness reduced to 2.5 mm and a pitch enlarged by two, corresponding to an improved parameter *λ* of 1.2 close to the optimal a value of 4/3 given by the model. Protocol 5 reduced *z*‐axial PVE and thereby increased the CNR, even with a lower CDTI_vol_. This shows that an optimal slice thickness may avoid increasing the dose in pursuit of a better detection. In clinical practice, orders of magnitude of lesions length have to be roughly estimated and be balanced against the choice of slice thicknesses for protocols optimization. We would for example recommend the use of a characteristic length $L=\frac{\pi }{4}d$ for spherical lesions of diameter *d*, giving an optimal slice thickness *T* ≅ 0.6*d* for nonoverlapping slices. The choice of a slice spacing of half the slice width would optimize the tradeoff between detectability and dose, and increase the optimal slice thickness to *T* ≅ 0.8*d*. Clinical experiments on human subjects could further validate the model for images with complex anatomical features and anatomical noise. This may be the scope of a future work that simulates various tumor shapes in tissue‐like surroundings.

Nevertheless, the analytical model may be easily applied for a complicated clinical task such as liver lesion detection. For example, Soo et al^8^ found that a slice thickness of 5 mm allowed a better detection, by radiologists, of lesions <5 mm in diameter than 2.5, 7.5, and 10 mm slices. *Z*‐axial PVE dramatically decreased the detection rate from 95% for 5 mm slices to 65% and 42% for the thicker 7.5‐ and 10‐mm slices, respectively, while an increase in image noise on the thinner 2.5‐mm slices decreased detection to 90%. An optimal slice thickness is therefore expected between 2.5 and 5 mm for lesions ~5 mm in diameter. Our model applied to this task gives an optimal slice thickness of *T* ≅ 0.6*d* = 3 mm for spherical lesions of diameter 5 mm (for nonoverlapping slices), in agreement with the results of Soo et al's study. The choice of a slice spacing of half the slice width would increase the optimal slice thickness to *T* ≅ 0.8*d* = 4 mm and optimize the ratio detectability / dose. A slice spacing of half the slice width maximizes the expected detectability, and allows a decrease in dose, but with tradeoff of increased image processing time, time for images review, and data storage requirements. The model may thus reasonably help clinical medical physicists optimize reconstruction parameters in cross‐sectional imaging protocols, and maximize the balance between detectability and dose for subslice objects.

## 5 Conclusion

An analytical model for the average statistical effect of *z*‐axial PVE on contrast‐to‐noise ratio (CNR) and detection was developed, and allowed to determine the optimal slice thickness and spacing for a given detection task. *Z*‐axial PVE was confirmed to significantly degrade the detection of thin objects in CT imaging, causing a loss in CNR. The analytical model provides an understanding relationship between axial resolution, noise, and dose that takes into account the expected contrast falloff due to *z*‐axial PVE. The averaged contrasts and CNR measured on the slices for different object sizes and positioning were in a good agreement with the model. The model allows knowledgeable selection of the reconstruction parameters (slice thickness and spacing) that, for a given *z*‐axial PVE, are required to restore the CNR to values that maximize the tradeoff between resolution and noise, or minimize the dose for a given object detection task. This model will contribute to help optimize CT protocols.
